# Retraction: Ammonia scavenger restores liver and muscle injury in a mouse model of non-alcoholic steatohepatitis with sarcopenic obesity

**DOI:** 10.3389/fnut.2025.1730980

**Published:** 2025-10-27

**Authors:** Frontiers Editorial Office

The journal retracts the March 17 2022 article cited above.

Following concerns regarding the originality of the cited article, an investigation was conducted in accordance with Frontiers' policies. The investigation validated the main points of the complaint, and in particular that it is unacceptably similar to a previously published article by Wang et al. in the *Journal of Practical Hepatology* (1), and the article is therefore retracted.

This retraction was approved by the Chief Editors of Frontiers in Nutrition and the Chief Executive Editor of Frontiers. The authors do agree with the retraction.

Frontiers would like to thank the users on PubPeer for bringing the published article to our attention.
